# Mutations in the *PLEKHG5* gene is relevant with autosomal recessive intermediate Charcot-Marie-Tooth disease

**DOI:** 10.1186/1750-1172-8-104

**Published:** 2013-07-12

**Authors:** Hyeon Jin Kim, Young Bin Hong, Jin-Mo Park, Yu-Ri Choi, Ye Jin Kim, Bo Ram Yoon, Heasoo Koo, Jeong Hyun Yoo, Sang Beom Kim, Minhwa Park, Ki Wha Chung, Byung-Ok Choi

**Affiliations:** 1 Department of Neurology, School of Medicine, Ewha Womans University, 911-1 Mokdong, Yangcheon-ku, Seoul 158-710, Korea; 2 Department of Biological Science, Kongju National University, 182 Sinkwan-dong, Gongju 314-701, Korea; 3 Department of Pathology, School of Medicine, Ewha Womans University, Seoul, Korea; 4 Department of Radiology, School of Medicine, Ewha Womans University, Seoul, Korea; 5 Department of Neurology, Kyung Hee University, College of Medicine, Seoul, Korea; 6 Department of Microbiology, Ewha Womans University, Seoul, Korea

**Keywords:** Charcot-Marie-Tooth disease (CMT), Lower motor neuron disease (LMND), *Pleckstrin homology domain-containing*, *Family G member 5*(*PLEKHG5*), Exome, Neuropathy

## Abstract

**Background:**

Mutations in the *Pleckstrin homology domain-containing, family G member 5* (*PLEKHG5*) gene has been reported in a family harboring an autosomal recessive lower motor neuron disease (LMND). However, the *PLEKHG5* mutation has not been described to cause Charcot-Marie-Tooth disease (CMT).

**Methods:**

To identify the causative mutation in an autosomal recessive intermediate CMT (RI-CMT) family with childhood onset, whole exome sequencing (WES), histopathology, and lower leg MRIs were performed. Expression and activity of each mutant protein were analyzed.

**Results:**

We identified novel compound heterozygous (p.Thr663Met and p.Gly820Arg) mutations in the *PLEKHG5* gene in the present family. The patient revealed clinical manifestations of sensory neuropathy. Fatty replacements in the distal lower leg muscles were more severe than in the thigh muscles. Although the symptoms and signs of this patient harboring slow nerve conduction velocities suggested the possibility of demyelinating neuropathy, a distal sural nerve biopsy was compatible with axonal neuropathy. Immunohistochemical analysis revealed that the patient has a low level of PLEKHG5 in the distal sural nerve and an in vitro assay suggested that the mutant proteins have a defect in activating the NF-κB signaling pathway.

**Conclusions:**

This study identifies compound heterozygous *PLEKHG5* mutations as the cause of RI-CMT. We suggest that PLEKHG5 might play a role in the peripheral motor and sensory nervous system. This study expands the phenotypic spectrum of *PLEKHG5* mutations.

## Background

Mutation in *Pleckstrin homology domain-containing, family G member 5* (*PLEKHG5*) has been reported only in a family with lower motor neuron disease (LMND) with childhood onset [1]. LMND, which is a large group of clinically and genetically heterogeneous disorders, is usually diagnosed by electrophysiological or histological evidence of muscle denervation without sensory neuropathy [2,3]. Except for *PLEKHG5*, many other causative genes have been reported in LMNDs: *SMN1* and *DYNC1H1*in each autosomal recessive (AR) and dominant spinal muscular atrophy (SMA: MIM 253300 and 158600), *HSPB1*,*HSPB8*, and *HSPB3*in distal hereditary motor neuronopathy type 2 (dHMN2: MIM 608634, 158590 and 613376), *GARS, BSCL2* and *REEP1* in dHMN5 (MIM 600794 and 614751), *IGHMBP2* in dHMN6 (MIM 604320), *DCTN1* and *SLC5A7* in dHMN7 (MIM 607641 and 158580), *ATP7A* in X-linked dHMN and SMA (MIM 300489), and *SETX* in dHMN with pyramidal sign (MIM 602433) [2-11]. The affected individuals harboring a *PLEKHG5* mutation presented with severe generalized distal and proximal muscle weakness and atrophy, but normal sensation; therefore, such mutations are often classified as a cause of dHMN [12] or generalized SMA [1]. However, there has been no report of a *PLEKHG5* mutation relevant to Charcot-Marie-Tooth disease (CMT), which is also called hereditary motor and sensory neuropathy (HMSN).

CMT is a clinically and genetically heterogeneous disorder of the peripheral motor and sensory nervous system [13]. CMT has been reported to be associated with more than 50 causative genes or loci [14]. Based on nerve biopsy and neurophysiology, CMT falls into two main subtypes, a demyelinating form (CMT1), and an axonal form (CMT2) [15]. An intermediate group exists with nerve conduction velocities, which overlaps the two main groups, and is subdivided into dominant intermediate (DI-CMT) and recessive intermediate CMT (RI-CMT) by its pattern of inheritance [16]. Intermediate forms of CMT may affect both axons and Schwann cells [17,18].

*PLEKHG5* is predominantly expressed in the peripheral nervous system, and the protein contains a DH (Dbl homology)- PH (Pleckstrin homology) motif, which is known as the minimal unit for the nucleotide exchange-promoting function of guanine nucleotide exchange factors (GEFs) [19]. PLEKHG5 protein has been suggested to have a role in the activation of the RhoA exchange factor and NF-κB signaling pathway [20], and it thus appears to be involved in neuronal cell differentiation. It is suggested that both loss of function in the NF-κB transduction pathway and aggregate formation of mutant PLEKHG5 might contribute to neurotoxicity [1,2].

In this study, we investigated a Korean RI-CMT family with childhood onset, and identified novel compound heterozygous mutations of *PLEKHG5*. To our knowledge, this is the first report of a *PLEKHG5* mutation with motor and sensory neuropathy.

## Subjects and methods

### Patients

This study enrolled 248 individuals in 160 unrelated families with variable types of CMT neuropathy. They were referred by their primary physicians or neurologists and selected negative for *PMP22* duplication and major CMT gene mutations during the period 2003–2012. This study also included 300 healthy controls. Informed consent was obtained from all participants according to the protocol approved by the Institutional Review Board for EwhaWomans University, Mokdong Hospital (ECT 11-58-37).

### DNA preparation and prescreening for CMT genes

DNA was purified from blood using a QIAamp blood DNA purification kit (Qiagen, Hilden, Germany). Patient samples were prescreened for 17p12 duplication, which is a major genetic cause of demyelinating CMT, by using hexaplex microsatellite PCR [21]. Sequencing was also determined for the coding exons of major CMT-relevant genes, including *MPZ*, *PMP22*, *GJB1*, and *MFN2*.

### Exome sequencing and identification of causative mutations

Exome sequencing was performed for the proband of FC307 family according to Lee et al. [22]. Functionally significant variants (missense, nonsense, exonicindel and splicing site variants) were first selected from WES data, and then novel or uncommon variants (MAF ≤0.01) registered in the dbSNP137 [23] and 1000 Genomes database [24] were chosen. Homozygous or compound heterozygous mutations were finally selected. Candidate variants were confirmed by Sanger’s sequencing method using an ABI3130XL automatic genetic analyzer (Applied Biosystems, Foster City, CA). The underlying cause was determined by the presence of mutation only in the patient within the family and the absence in 300 controls. Conservation analysis of protein sequences was performed using MEGA5 version 5.05 software [25].

### Clinical and electrophysiological assessments

**Figure 1:**
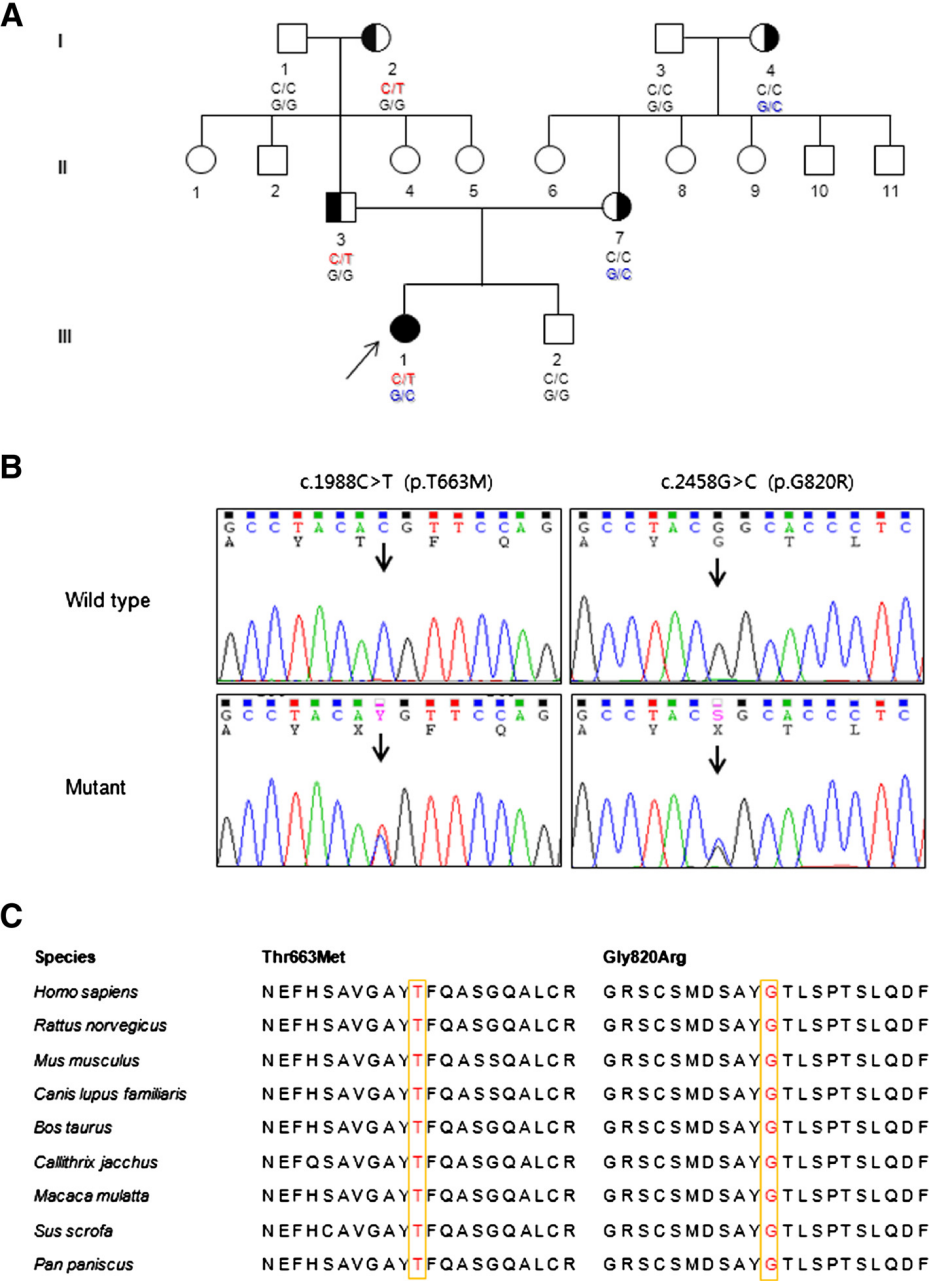
**Pedigree, sequencing chromatograms and conservation in the FC307 family with compound heterozygous *PLEKHG5 *mutations.** (**A**) Pedigree. Open symbols indicate unaffected individuals and the filled symbol indicates the affected individual. Half-filled symbols indicate carriers possessing one mutant allele. The arrow indicates the proband. Genotypes of both *PLEKHG5* mutations are indicated at below each examined individual. (**B**) Sequencing chromatograms. Vertical arrows indicate the mutation site. (**C**) Conservation analysis of amino acid sequences. The analysis was conducted using MEGA5 version 5.05 software. Both mutation sites were well conserved across species.

**Figure 2:**
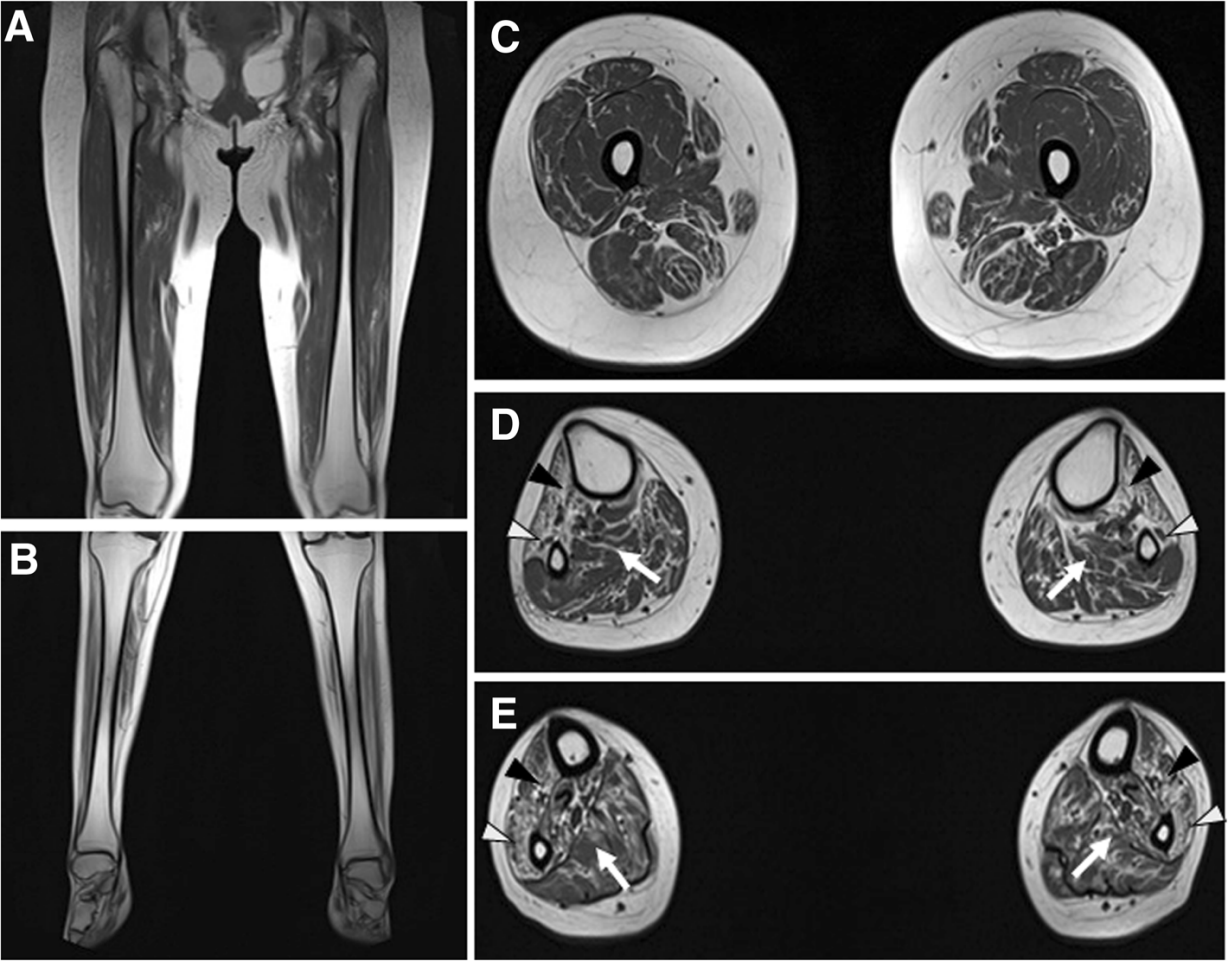
**T1-weighted MRI of the proband (III-1).** (**A**, **B**) Coronal images of thigh (**A**) and lower leg (**B**). T1-weighted coronal leg MRIs of lower extremities demonstrated more severe fatty replacement and muscle atrophies in the lower leg than did those of the thigh. (**C**) Axial image of the middle thigh. Vastus lateralis, semimembranous muscles showed moderate to severe fatty replacements, but they revealed more muscles than fat. However, sartorius, gracilis, biceps femoris, and rectus femoris muscles revealed fatty streaks. (**D**, **E**) Axial images at the upper third (**D**) and lower third calf (**E**). T1-weighted images revealed predominantly severe involvement of anterior (black arrowhead; anterior tibialis, extensor hallucis longus and digitorum longus) and lateral (white arrowhead; peroneus longus and brevis) compartment muscles, and superficial and deep posterior compartment (arrow; soleus and tibialis posterior) muscles showed mild involvement.

**Figure 3:**
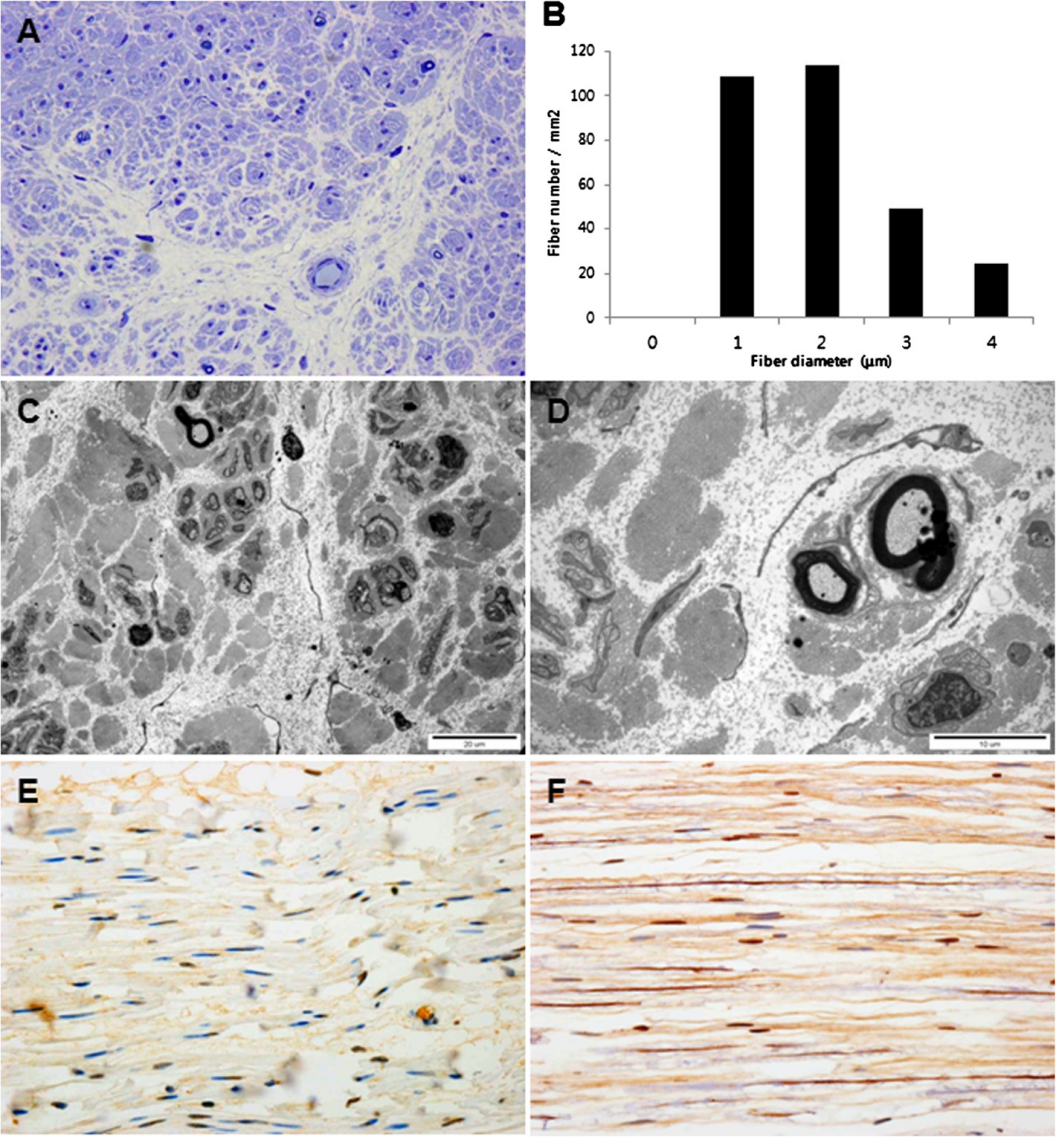
**Histopathological findings of the distal sural nerve of the patient.** (**A**) Toluidine blue-stained semi-thin transverse section revealed loss of large MFs and remaining medium- and small-sized MFs with rarely noted regenerating axonal clusters and unmyelinated axons with suggestive clustering and atrophy. (**B**) Histogram showing unimodal distribution pattern of myelinated fibers. (**C** and **D**) Electron micrographs showed axonal atrophy, rare axonal clustering, occasional focal folding of myelin, and increased collagen deposition in the endoneurium. (**E**) Immunohistochemical study with anti-PLEKHG5 antibody showed a focal positive reaction in the patient’s Schwann cell nuclei. (**F**) Comparison with diffuse strong positive reaction in axons and Schwann cell nuclei in the control case (original magnification: **A**, ×400; **C**, ×3000, **D**, ×8000, **E** and **F**, ×400).

**Figure 4:**
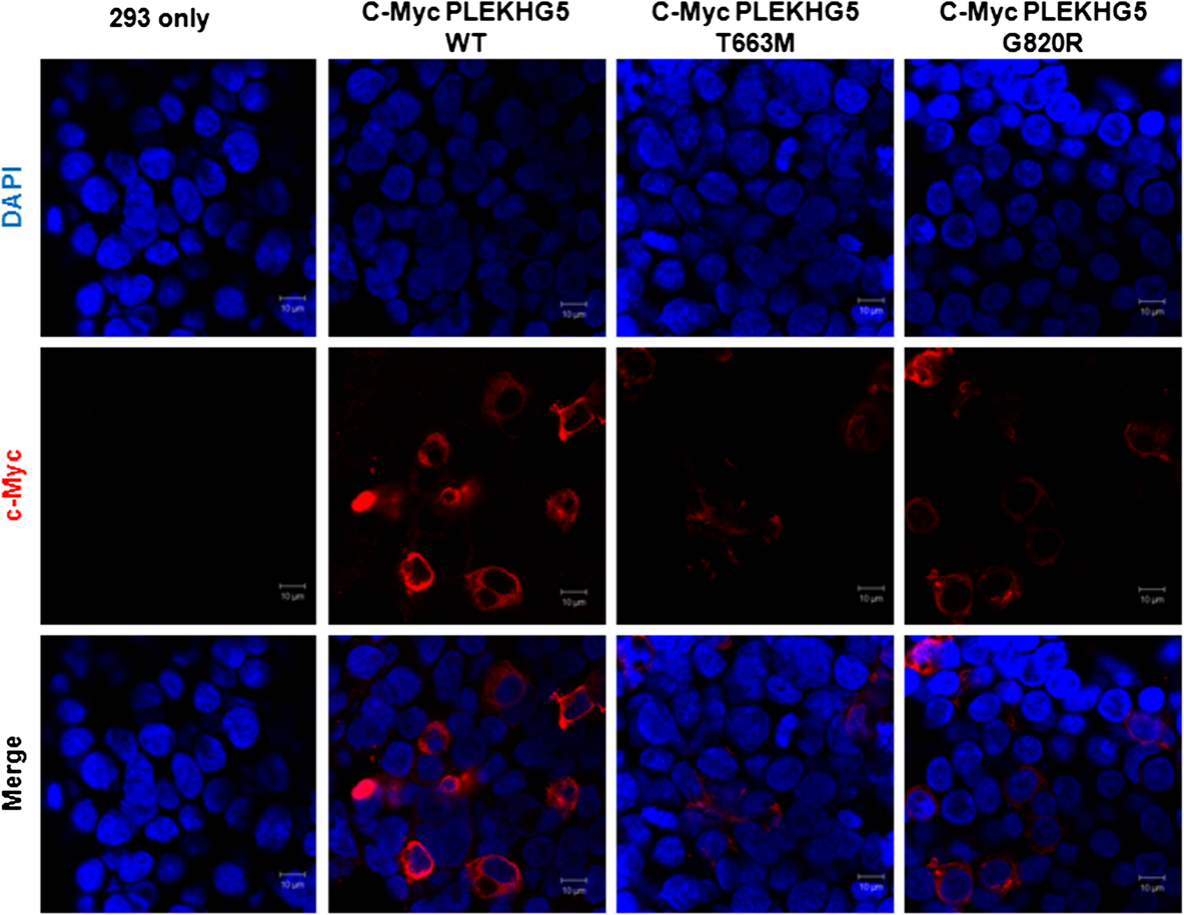
**Cellular localization of wild-type and mutant PLEKHG5 proteins.** Immunocytochemical analysis reveals that wild-type and mutant PLEKHG5 proteins were localized in the cytoplasm and there was no distinctive aggregation of any of the proteins. HEK293 cells transfected with wild-type or mutant PLEKHG5-expressing vectors were stained with DAPI (nucleus) and c-Myc antibody.

The proband (Figure 1A, III-1), her parents (Figure 1A, II-3, and −7), and grandparents (Figure 1A, I-1, -2, -3, and −4) were examined for motor and sensory impairments, deep tendon reflexes, and muscle atrophy. The strength of flexor and extensor muscles was assessed manually using the medical research council (MRC) scale. Physical disabilities were assessed using a CMT neuropathy score (CMTNS) [26]. Sensory impairments were assessed in terms of the level and severity of pain, temperature, vibration, and position. Age at onset was determined by asking patients for the age when symptoms, i.e., distal muscle weakness, foot deformity, or sensory change, first appeared.

Nerve conduction studies were performed with a surface electrode. Motor nerve conduction velocities (MNCVs) of the median and ulnar nerves were determined by providing stimulation at the elbow and wrist while recording compound muscle action potentials (CMAPs) over the abductor pollicis brevis and adductor digiti quinti, respectively. In the same manner, the MNCVs and CMAPs of the peroneal and tibial nerves were determined. Sensory nerve conduction velocities (SNCVs) and action potentials (SNAPs) were obtained from the median, ulnar, and sural nerves. Needle electromyography (EMG) was performed for bilateral proximal and distal limb muscles. The patient and her parents underwent tests for visually evoked potentials and brainstem auditory evoked potentials.

### MRIs of the brain, hip, thigh and lower leg

The proband was studied by examining MRIs of the brain, hip, thigh, and lower leg, using a 1.5-T system (Siemens, Erlangen, Germany). Lower limb imaging was obtained in axial [field of view (FOV) 24–32 cm, slice thickness 6 mm, and slice gap 0.5–1.0 mm] and coronal planes (FOV 38–40 cm, slice thickness 4–5 mm, slice gap 0.5–1.0 mm). The following protocol was used: T1-weighted spin-echo (SE) (TR/TE 570–650/14–20, 512 matrices), T2-weighted SE (TR/TE 2800–4000/96–99, 512 matrices), and fat-suppressed T2-weighted SE (TR/TE 3090–4900/85–99, 512 matrices).

### Histopathological examination

The distal sural nerve of patient (III-1) was biopsied at 19 years of age. The density of myelinated fibers (MFs), axonal diameter, and myelin thickness were determined from the semi-thin transverse sections using a computer-assisted image analyzer (AnalySIS; Soft Imaging System, Münster, Germany). Ultrathin cut samples (60–65 nm) were contrasted with uranyl acetate and lead citrate for electron microscopy (H-7650, Hitachi, Japan). An immunohistochemical study was performed with anti-PLEKHG5 antibody (N-15: sc-130100, 1:50 dilution, Santa Cruz Biotech, Santa Cruz, CA) and the findings were compared with a control case (female, 39 years old).

### Cloning, expression and reporter assay for *PLEKHG5*

*PLEKHG5* (BC015231), which is fused with a c-*myc* epitope, was obtained from Capital Biosciences (Rockville, MD). Site-directed mutagenesis was performed to generate mutant *PLEKHG5* (p.Thr663Met and p.Gly820Arg) using a QuickChange Site-directed mutagenesis kit (Stratagene, La Jolla, CA). DNA transfections into HEK293 cells were performed using each DNA and Lipofectamine 2000 (Invitrogen, Carlsbad, CA). Protein expression was analyzed by immunocytochemistry using anti-Myc antibody (Abcam, Cambridge, UK) after transfection of each vector. Reporter assay for NF-κB was achieved using a reporter vector, pNF-κB-Luc-reporter (from Dr. Kim HT, Sungkyunkwan University, Korea), and a luciferase assay system (Promega, Madison, WI).

## Results

### Identification of compound heterozygous mutations in *PLEKHG5*

To identify the disease-associated genetic defect of our patient, we performed exome sequencing of the affected individual (Table 1). We identified novel compound heterozygous mutations, c.1988C > T (p.Thr663Met, paternal origin) and c.2458G > C (p.Gly820Arg, maternal origin) in the *PLEKHG5* gene in a Korean CMT family (family ID: FC307) (Figure 1A and 1B). The mutations of her parents were inherited from their mothers, respectively (Figure 1A). Neither mutation has been reported in dbSNP137 or the 1000 Genomes database. In the 300 Korean controls, the p.Thr663Met mutation was not found, while the p.Gly820Arg was found in three control samples. Both mutation sites were well conserved across species (Figure 1C). *In silico* analysis using PolyPhen 2 software [27] predicted that both mutations may affect protein function with scores of 0.993 (p.Thr663Met) and 1.000 (p.Gly820Arg). In addition to the compound heterozygous mutations in *PLEKHG5*, several other variants were identified in the more than 50 CMT-associated genes (Table 2). However, no variant was considered to be causative, because all the variants were polymorphic; moreover, they did not fit the recessive inheritance characteristic.

**Table 1:** Whole exome sequencing analysis in the affected individual (III-1)

**Items**	**FC307 (III-1)**
Total sequencing yields (Gbp)	6.5
% Mappable reads (/total reads)	94.0
% Coverage of target regions (more than 10X)	93.4
Mean read depth of target regions	88.5X
Total observed SNPs	57,356
Total observed Indels	9,592
Filtering	
Coding SNPs	19,700
Coding Indels	526
Functionally significant variants^a^	9,136
Functionally significant variants in CMT genes	30

CMT, Charcot-Marie-Tooth disease; SNP, single nucleotide polymorphism.

^a^Nonsynonymous variants include splicing site, frameshift, stop gain, and stop loss.

**Table 2:** Polymorphic nonsynonymous or splicing site variants in CMT-associated genes from the exome data

**Gene**	**RefSeq**^**a**^	**Nucleotide change**^**b**^	**Amino acid change**	**SNP quality**	**Read depth**	**Mutant allele no.**	**dbSNP137/1000 Genomes**^**c**^	**Mutant allele frequencyin Korean controls**
*KIF1B*	NM_015074	c.2192A > G	p.N731S	155	56	31	rs117525287/0.01	0.042
*FIG 4*	NM_014845	c.1090A > T	p.M364L	177	191	87	rs2295837/0.10	0.268
		c.1961T > C	p.V654A	225	128	63	rs9885672/0.37	0.377
*GARS*	NM_002047	c.124C > G	p.P42A	51	14	8	rs1049402/0.67	0.380
*ARHGEF10*	NM_014629	c.824G > A	p.R275H	225	124	58	rs145821459/<0.01	0.024
*NEFL*	NM_006158	c.1413delC	p.P471fs	105	115	106	rs11300136/–	0.697
*IKBKAP*	NM_003640	c.3473C > T	p.P1158L	108	103	44	rs1538660/0.22	0.301
		c.3214T > A	p.C1072S	225	67	30	rs3204145/0.22	0.310
		c.2490A > G	p.I830M	135	130	70	rs2230794/0.08	0.151
		c.2446A > C	p.I816L	82	171	154	rs2230793/0.29	0.310
		c.2294G > A	p.G765E	91	77	72	rs2230792/0.28	0.310
*LRSAM1*	NM_138361	c.952A > G	p.N318D	157	40	19	rs1539567/0.74	0.690
*SETX*	NM_015046	c.7834A > G	p.S2612G	151	47	22	rs3739927/0.16	0.403
		c.7759A > G	p.I2587V	225	41	19	rs1056899/0.51	0.781
		c.5563A > G	p.T1855A	95	70	40	rs2296871/0.41	0.747
		c.4156A > G	p.I1386V	225	113	44	rs543573/0.59	0.273
		c.3576T > G	p.D1192E	225	71	25	rs1185193/0.66	0.293
		c.1979C > G	p.A660G	102	50	23	rs882709/0.21	0.387
*IGHMBP2*	NM_002180	c.602T > C	p.L201S	156	83	78	rs560096/0.70	0.566
		c.2636C > A	p.T879K	166	37	29	rs17612126/0.23	0.355
		c.2782G > A	p.E928K	132	16	14	rs2275996/0.03	0.106
*MTMR2*	NM_016156	c.8A > C	p.K3T	65	12	6	rs3824874/0.28	0.284
*WNK1*	NM_213655	c.3922A > C	p.T1308P	33	42	13	rs956868/0.85	0.051
		c.5273G > C	p.C1758S	222	55	53	rs7955371/0.99	1.000
		c.6180G > T	p.M2060I	162	123	55	rs12828016/0.39	0.299
*SEPT9*	NM_006640	c.1672A > G	p.M558V	222	13	13	rs2627223/0.92	0.937
*PRX*	NM_181882	c.3394G > A	p.G1132R	115	26	24	rs268674/0.96	1.000
*ATP7A*	NM_000052	c.4048G > A	p.E1350K	222	201	182	rs4826245/1.00	1.000

^a^ GenBank registration number of reference sequence.

^b^ cDNA numbering was achieved with +1, corresponding to the A of the ATG initiation codon.

^c^ GenBank registration no./allele frequencies in the 1000 Genomes database (−: unreported).

### Clinical manifestations

The clinical manifestations of the present patient and the previously described patients with *PLEKHG5* mutations are shown in Table 3. The proband, a 19-year-old woman, was the first child of healthy nonconsanguineous Korean parents. On neurological examination and after electrophysiological studies, the parents did not show any abnormalities. The proband was born at full term and the perinatal history was unremarkable. Early motor milestones were not delayed, and one year after her birth, she was able to walk. At age 8 years, she experienced frequent falling and noticed muscle weakness of the distal lower limbs. She started to walk with short leg braces at 16 years of age. Neurological examination at 19 years of age revealed muscle weakness and atrophy of bilateral distal muscles, predominantly at lower limbs rather than upper limbs. Bilateral pes cavus, steppage gait, and atrophic changes of intrinsic foot and calf muscles were noted, but scoliosis was not observed. Sensitivity to pinprick, touch, position, and vibration decreased. Vibration and position senses were more severely disturbed than pain and touch senses. Knee and ankle jerks were absent. No pyramidal or cerebellar signs were detected. CMTNS was 15. Elevated serum creatine kinase levels were revealed (246 μmol/L, reference value: <185 μmol/L).

**Table 3:** **Clinical manifestations of patients with *PLEKHG5 *mutations**

**Disease**	**Charcot-Marie-Tooth disease**	**Lower motor neuron disease**
Phenotype	Motor and sensory neuropathy	Motor neuropathy
Origin	Korean (Asian)	Mali (African)
Mutation	Compound heterozygous missense	Homozygous missense
Nucleotide change	c.1988C > T, c.2458G > C	c.1940T > C
Amino acid change	p.Thr663Met, p.Gly820Arg	p.Phe647Ser
Age at onset	8 years	2–11.5 years
Symptom at onset	Distal muscle weakness of lower limbs	Proximal muscle weakness and early involvements of foot and hand
Muscle weakness		
Upper limb, proximal	No	Yes
Upper limb, distal	Yes	Yes
Lower limb, proximal	Yes	Yes
Lower limb, distal	Yes	Yes
Muscle atrophy	Proximal < distal	Proximal = distal (generalized)
Sensory loss	Yes	No
Areflexia	Yes	Yes
Pyramidal sign	No	No
Bulbar symptom	No	No
Cranial neuropathy	No	No
Foot deformity	Yes	Yes
Scoliosis	Yes	Yes
Respiratory dysfunction	No	Yes (60%)
Wheelchair bound	No	Yes (80%)
Nerve conduction study	Sensorimotor neuropathy	Motor neuropathy
Electromyography	Muscle denervation	Muscle denervation
Sural nerve biopsy	Severe loss of myelinated fibers (297/mm^2^, normal: 9,800/mm^2^)	Normal
References	This study	Maystadt et al. (2006) [1]

### Sensory and motor neuropathies

Data from the nerve conduction study are summarized in Table 4. Median MNCVs ranged from 24.7 m/s to 29.3 m/s. The results also revealed prolonged motor latencies, and no motor responses were elicited by stimulation of peroneal and tibial nerves at 19 years of age. SNAPs on bilateral sural nerves were absent on all electrophysiological studies (at 14, 15, 16 and 19 years old). In the upper limb, SNCVs and SNAPs were decreased on bilateral median and ulnar nerves. Needle EMG showed a neurogenic pattern of muscle degeneration. Visually evoked potentials and brainstem auditory-evoked potentials were normal.

**Table 4:** **Electrophysiological studies in the patient (III-1) with a novel compound mutations in the *PLEKHG5 *gene**

**Items**	**Determined values**				**Normal value**
Age at exam (years)	14	15	16	19	
Side	Right	Right	Right	Right	
Median nerve					
TL (ms)	**6.1**	**5.4**	**5.5**	**7.0**	<3.9
CMAP (mV)	8.2	9.5	9.6	6.4	>6.0
MNCV(m/S)	**25.3**	**24.7**	**25.6**	**29.3**	>50.5
Ulnar nerve					
TL (ms)	**4.9**	**4.9**	**4.9**	**5.7**	<3.0
CMAP (mV)	9.7	10.2	10.0	10.0	>8.0
MNCV(m/S)	**25.0**	**22.4**	**20.4**	**25.0**	>51.1
Peroneal nerve					
TL (ms)	**A**	**A**	**A**	**A**	<5.3
CMAP (mV)	**A**	**A**	**A**	**A**	>1.6
MNCV(m/S)	**A**	**A**	**A**	**A**	>41.2
Tibial nerve					
TL (ms)	**7.2**	**6.0**	**7.4**	**A**	<5.4
CMAP (mV)	**0.6**	**0.3**	**0.2**	**A**	>6.0
MNCV(m/S)	**19.2**	**15.8**	**13.4**	**A**	>41.1
Median sensory nerve					
SNAP (μV)	**6.5**	**4.8**	**7.8**	**8.0**	>8.8
SNCV (m/s)	**27.0**	**25.9**	**27.3**	**25.6**	>39.3
Ulnar sensory nerve					
SNAP (μV)	**7.4**	**6.9**	**5.0**	**4.5**	>7.9
SNCV (m/s)	**23.4**	**21.9**	**22.3**	**23.7**	>37.5
Sural nerve					
SNAP (μV)	**A**	**A**	**A**	**A**	>6.0
SNCV (m/s)	**A**	**A**	**A**	**A**	>32.1

Bold character indicates abnormal values. A, absent potentials; TL, terminal latency; CMAP, compound muscle action potential; MNCV, motor nerve conduction velocity; SNAP, sensory nerve action potential; SNCV, sensory nerve conduction velocity.

### Fatty replacement of lower limb muscles

Brain and hip MRIs were normal. However, thigh and lower leg MRIs of the patient demonstrated hyperintense signal abnormalities. T1-weighted images showed severe muscle atrophy and fatty replacement in the lower leg muscles compared with the thigh, which is compatible with a hypothesis of length-dependent axonal degeneration (Figure 2A and 2B). At the thigh level, fatty replacement of the vastus lateralis and semimembranous muscles was observed, but more muscle remained than fat. Sartorius, gracilis, biceps femoris, and rectus femoris muscles contained some fatty streaks (Figure 2C). We could observe a distinct pattern of muscle involvement; lower leg MRI showed selective and predominant involvement of anterior and lateral compartment muscles; however, superficial and deep posterior compartment muscles revealed mild manifestations (Figure 2D and 2E).

### Histopathological findings

Semi-thin transverse sections showed loss of large- and medium-sized MFs, with small MFs remaining (Figure 3A). The number of MFs was reduced (297/mm^2^) compared with a control (20-year-old female, 9,800/mm^2^) (Figure 3B). The range (1.2-4.6 μm) and average (2.4 μm) MF diameter were also reduced compared with the control (2–12.5 μm and 4.5 μm, respectively). The distribution pattern of MF diameter was unimodal and composed of 75% of <3 μm MFs. In addition, MF% area and g-ratio (axonal diameter/MF diameter) were also reduced. The MF% area in this case was 0.2% (normal distal sural nerve in 20-year-old female: 25.2%). The range and average of g-ratio (axonal diameter/MF diameter) were 0.38-0.73 and 0.56 ± 0.09, respectively (mean g-ratio at age 21–50 years old: 0.66). The g-ratio >0.7 (abnormally thin myelin sheath) consisted of 10% of MFs and g-ratio <0.4 (abnormally thick myelin sheath) consisted of 1.7%. Electron microscopic examination revealed that the remaining small MFs showed occasional focal folding of myelin with very rare evidence of regeneration (clusters of regenerating fibers) (Figure 3C and 3D). The unmyelinated axons showed suggestive clustering (reinnervated Büngner bands with unmyelinated fibers) and atrophy. Endoneurial fibroblast proliferation and large amounts of collagen deposition were well documented.

### Expression of mutant proteins

The immunohistochemical study with anti-PLEKHG5 antibody showed a focal positive reaction in Schwann cell nuclei in this patient (Figure 3E) compared with a diffuse positive reaction in axons and Schwann cell nuclei in the control case (Figure 3F). To further investigate the impact of the mutant proteins in cellular function and localization, we generated mutants using a BC015231 clone as a template. Cellular localization of the proteins was analyzed after transfection of the genes into HEK293 cells. Overall, the proteins were localized in the cytoplasm and there was no distinctive aggregation of any of the three proteins (Figure 4).

**Figure 5:**
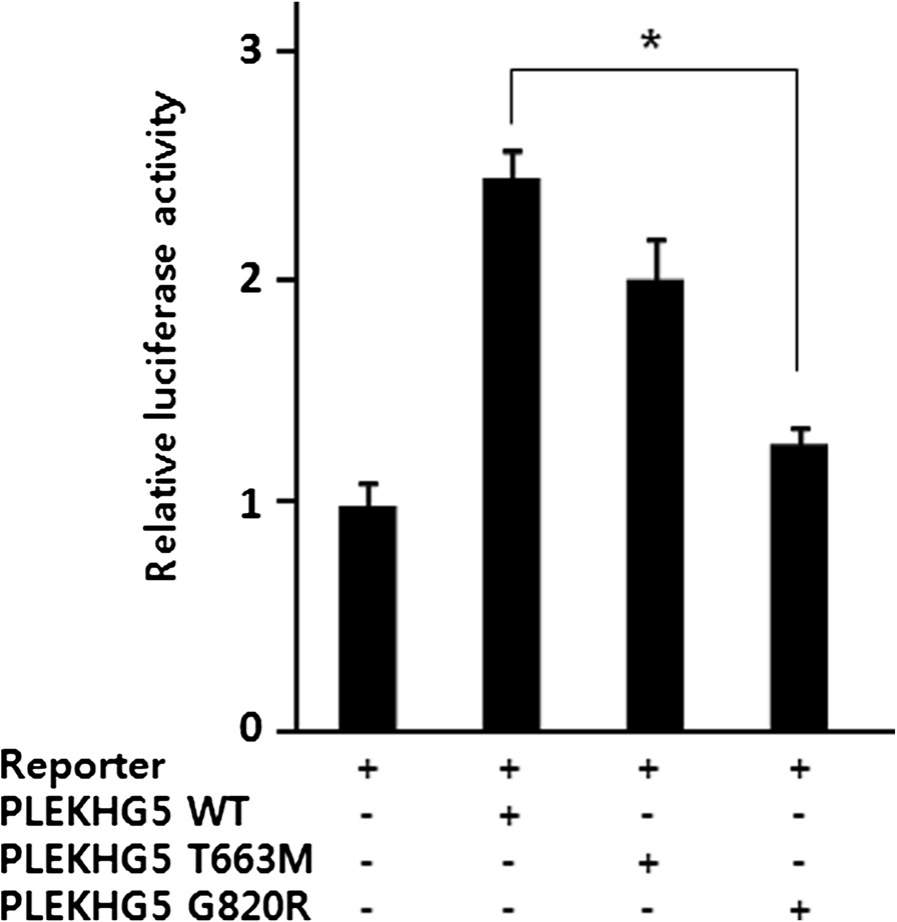
**Activities of wild-type and mutant PLEKHG5.** Compared with wild-type, mutant PLEKHG5 proteins have defect in the activation of NF-κB pathway. Luciferase activity measured in HEK293 cells, which were treated with pNF-κB-Luc-reporter and *PLEKHG5*-expressing vectors. Statistical significance was calculated using Student’s *t* test: *, *p* < .05.

The proteins were examined for ability to activate the NF-κB pathway using a luciferase assay. Transfection of wild-type PLEKHG5 significantly induced luciferase activity. However, elevated levels of the activity were reduced in mutant protein-expressing cells (Figure 5).

## Discussion

We have identified a pair of novel compound heterozygous mutations of *PLEKHG5* as the underlying cause of the disease in the CMT family with autosomal recessive inheritance. Although the sample size in this family was small, the mutations were in good agreement with affected or carrier members in the pedigree. These identified mutations have not been reported at either the CMT or LMND mutation database or in related papers. We believe that the identified *PLEKHG5* mutations are responsible for the CMT for the following reasons: (1) the patient harbors paternal p.Thr663Met and maternal p.Gly820Arg mutations, but no other family members or controls had both heterozygous mutations simultaneously, (2) amino acids at the mutation sites among different species are well conserved, and (3) exome sequencing revealed that no causative mutation of the many known CMT genes was found in the present family.

Sensory neuropathy is a useful discriminating factor between CMT and LMND [1,13]. LMND is characterized by motor nerve degeneration in the anterior horn cell of the spinal cord or the brainstem, and the diagnosis is confirmed by the evidence of muscle degeneration with abnormal motor conduction velocities without sensory neuropathy [1,3]. However, CMT involves not only motor, but also sensory neuropathies. The present patient showed clinical, electrophysiological, and pathological evidence of sensory nerve involvement; therefore, she was diagnosed as having CMT.

It is interesting that the symptoms and signs of this patient such as sensory loss and slow nerve conduction velocities suggested the possibility of demyelinating neuropathy; however, the distal sural nerve biopsy revealed similarities with axonal neuropathy harboring absence of medium- and large-sized MFs. Absence of large MFs could explain the slow nerve conduction velocities and action potentials in this patient. The intermediate form of CMT has been observed in cases with median MNCVs between 25 and 45 m/s, and in our patient, median MNCVs were at the lower extreme of the range [16-18,28]. By neurophysiological and neuropathological criteria, the current case was consistent with the RI-CMT.

Currently, information on the molecular function and disease-associated mechanism of *PLEKHG5* is limited: only one homozygous mutation, p.F647S, was reported as a causative for autosomal recessive LMND. The site locates within the PH domain and the mutation was suggested to deteriorate the function on activation of NF-κB signaling pathway. Thr663 lies within the PH domain as does the previously reported Phe647, while Gly820 does not belong to any specific domain. Our in vitro data also suggest that these patients might have a defect in activation of the NF-κB signaling pathway. According to immunohistochemical data from the patient and immunocytochemical study using cloned mutants, the expression levels of the mutant proteins are lower than that of wild-type protein. By contrast with the p.F647S mutation, however, there was no indication of intracellular aggregation of current mutants. Therefore, these data imply that the low level of expression and NF-κB activation might induce peripheral neuropathy in the patient.

The PH domain of GEFs for Rho-related GTPase plays a critical role in the spatial organization of major signaling pathways, through their interaction with lipids (especially with phosphoinositides) and proteins [29]. NF-κB signaling could be included in those pathways in conjunction with the function of RhoA in triggering the translocation of NF-κB into the nucleus, because PLEKHG5 is known as an activator of RhoA exchange factor. As well as its well-known function in immune and inflammatory responses, the NF-κB signaling pathway is also involved in the development and activity of the nervous system. Particularly in the peripheral nervous system, NF-κB stimulation has been shown to have essential roles in the differentiation and myelinating programs as well as Schwann cell regeneration [30]. However, several questions regarding the pathological mechanism in our patient remain to be answeredabout the PH domain defect of the PLEKHG5 protein and congenital dysfunction in peripheral nervous system via NF-κB signaling pathway. Other human neurodegenerative disease-related genes sharing a PH/RhoGEF domain, such as *DNM2, SBF2*, and *ALS2,* could provide important clues for these elusive questions [31-33].

## Conclusions

We identified novel compound heterozygous *PLEKHG5* mutations as the underlying cause of RI-CMT. In the present case, nerve conduction studies showed slow motor and sensory nerve conduction, and the distal sural nerve revealed marked loss of myelinated fibers. Our findings significantly expand the phenotype of *PLEKHG5* mutation; therefore, genetic screening of *PLEKHG5* should be considered in not only LMND but also RI-CMT families. We suggest that PLEKHG5 plays an important role in both the peripheral motor and sensory nervous systems.

## Abbreviations

CMAP: compound muscle action potential; CMT: Charcot-Marie-Tooth disease; CMTNS: CMT neuropathy score; EMG: electromyography; dHMN: distal hereditary motor neuronopathy; HMSN: hereditary motor and sensory neuropathy; LMND: lower motor neuron disease; MNCV: motor nerve conduction velocities; PLEKHG5: *Pleckstrin homology domain-containing, family G member 5*; SMA: spinal muscular atrophy; SNAP: sensory nerve action potentials; SNCV: sensory nerve conduction velocities; WES: whole exome sequencing.

## Competing interests

The authors declare that they have no competing interests.

## Authors’ contributions

KHJ and CB-O: study concept and design; HYB, CYR, PM, KH, CKW and CB-O: acquisition of data; HYB, PJ-M, KYJ, YBR, and KH: analysis and interpretation of data; KHJ and PJ-M: drafting of the manuscript; KH, CKW, and CB-O: critical revision of the manuscript for important intellectual content; KYJ, YBR and PJ-M: administrative, technical, and material support; CKW, and CB-O: provision of funding and study supervision. All authors read and approved the final manuscript.
